# Maternal Determinants of Human Milk Leptin and Their Associations with Neonatal Growth Parameters

**DOI:** 10.3390/nu18020192

**Published:** 2026-01-07

**Authors:** Yaiza Garro-Aguilar, Egoitz Astigarraga, Gabriel Barreda-Gómez, Olaia Martinez, Edurne Simón

**Affiliations:** 1 Research and Development Department, Amaltea Research, 48940 Leioa, Spain; ygarro001@ikasle.ehu.eus (Y.G.-A.); egoitz.astigarraga@imgpharma.com (E.A.); 2 GLUTEN3S Research Group, Department of Pharmacy and Food Sciences, University of the Basque Country UPV/EHU, 01006 Vitoria-Gasteiz, Spain; olaia.martinez@ehu.eus; 3 Research and Development Division, IMG Pharma Biotech, 48170 Zamudio, Spain; gabriel.barreda@imgpharma.com; 4 Nutrition and Food Safety Research Group, Bioaraba Health Research Institute, 01006 Vitoria-Gasteiz, Spain

**Keywords:** human breast milk, milk leptin, preterm delivery, maternal BMI, neonatal growth parameters, maternal determinants

## Abstract

**Introduction:** Human breast milk (HBM) is a critical source of nourishment for newborns, containing bioactive compounds that influence infant growth and metabolic programming. Among these compounds, leptin—a hormone primarily produced by adipocytes but also synthesized in the mammary gland—has gathered attention for its potential role in regulating energy balance and body weight. This study investigates the influence of maternal factors on HBM leptin concentrations and explores their associations with neonatal growth parameters. **Material and Methods:** 262 HBM samples were collected from healthy lactating mothers through Spanish Biobanks during the first six months postpartum. Data on maternal characteristics (body mass index (BMI), age, physical activity, parity, and delivery type) and neonatal measurements (weight, length, and head circumference) were collected. Leptin concentrations in skimmed HBM were measured using the ELISA technique (R&D Systems™, Minneapolis, MN, USA). Statistical analyses were conducted using R version 4.3.1 and MATLAB R2023a, with significance set at *p* < 0.05. **Results:** Leptin levels were highest in and declined over time, reaching a stable level after the first month of lactation. Preterm deliveries exhibited significantly higher leptin concentrations than term deliveries (0.42 vs. 0.07 ng/mL). Higher leptin levels were also observed in younger and primiparous mothers. Maternal BMI was positively associated with leptin concentration, with mothers who had elevated BMI showing higher levels than those with optimal BMI (0.36 vs. 0.05 ng/mL). Maternal physical activity was not associated with leptin concentrations in univariate analyses; although greater self-reported physical activity appeared associated with lower leptin concentrations in regression models, this finding should be interpreted cautiously and should not be considered evidence of an independent or consistent effect. Neonatal growth parameters (weight, length, and head circumference) were negatively correlated with HBM leptin concentrations. **Conclusions:** Our findings indicate that leptin levels in breast milk reflect both maternal metabolic status and neonatal characteristics and may represent a compensatory mechanism in preterm infants. HBM leptin levels are modulated by maternal BMI, age, parity, and delivery type, and are associated with neonatal growth parameters.

## 1. Introduction

The first months of life constitute a critical period for shaping long-term health, with human breast milk (HBM) playing a key role in this early developmental period. HBM is widely recognized as the optimal source of nutrition for newborns, due to its ability to support growth and development [1,2,3]. Beyond supplying essential nutrients, HBM contains a complex array of bioactive molecules that modulate key early-life processes, such as brain maturation, immune system development, and gastrointestinal function [4,5,6,7]. Among these bioactive components, leptin—a hormone primarily secreted by adipose-tissue and naturally present in breast milk—has attracted considerable interest for its involvement in appetite regulation, energy balance, and early growth patterns [8,9,10,11,12,13]. Acting as a metabolic signal, leptin has been proposed to be involved in satiety regulation and adiposity; however, evidence in humans remains largely observational [14].

Importantly, although leptin has been proposed as a mediator of early metabolic programming, most evidence in humans derives from observational studies. Therefore, reported associations between human milk leptin and infant outcomes should be interpreted as correlational rather than causal.

Leptin concentrations in HBM vary throughout lactation and show substantial interindividual differences among lactating mothers [15,16,17]. Maternal characteristics appear to be key contributors of this variability, with factors such as body composition, nutritional status, hormonal balance, as well as environmental and physiological conditions being associated the modulation of milk leptin levels [18,19].

However, evidence regarding leptin concentrations in preterm versus term human milk remains contradictory across the literature. While some studies report higher leptin levels in preterm milk, others describe lower concentrations or no significant differences when compared with term milk. These inconsistent findings reveal a clear knowledge gap concerning how gestational age at delivery influences leptin concentrations in human breast milk.

In Spanish cohorts, maternal characteristics such as BMI, age, parity, and delivery type have been shown to influence the composition of human breast milk, including mineral content, hormonal levels, and immune components [20,21,22]. These findings underscore the importance of considering obstetric and anthropometric factors when studying milk bioactive compounds and their potential impact on infant growth and development.

Overall, it is essential to understand how maternal traits influence leptin concentrations, as these variations may be relevant for understanding variability in early growth patterns, although long-term effects cannot be inferred from observational data [17].

This study aims to investigate the associations between maternal characteristics—including BMI, age, and physical activity level—on HBM leptin concentrations. Clarifying these relationships is crucial for developing targeted nutritional and public health strategies to optimize infant health and reduce the risk of later metabolic disorders.

## 2. Materials and Methods

### 2.1. Sample and Data Collection

All biological samples and associated donor data used in this study were provided by two Spanish Biobanks: Biobank Hospital Universitario Puerta de Hierro Majadahonda (HUPHM)/Instituto de Investigación Sanitaria Puerta de Hierro-Segovia de Arana (IDIPHISA) (PT17/0015/0020) and Biobank of the Aragon Health System (PT20/00112), both integrated within the Spanish National Biobanks Network. Samples collection and processing followed standardized procedures and were approved by the corresponding Ethics and Scientific Committees.

In addition, the present project was approved by the Ethics Committee for Human Research of Hospital Universitario Puerta del Hierro de Majadahonda (Madrid) (Ref: 57/723952.9/22, on 16 December 2022) and by the Research Ethics Committee of the Autonomous Community of Aragon (CEICA) (Ref: PI22/332, on 17 June 2022).

A total of 262 samples were collected from 98 healthy lactating mothers, meaning that some donors contributed multiple samples across different lactation stages, from the first postpartum day through the sixth month of lactation. All samples were stored at −80 °C until analysed.

Sample and data collection were performed simultaneously. Recorded maternal variables included physical activity level (assessed via categorical self-report; no validated questionnaire or standardized scale was used), parity, age, delivery type (preterm or term, with preterm defined as <37 weeks of gestation and term as ≥37 weeks), and body mass index (BMI), calculated as weight (kg/height (m)^2^). Neonatal parameters comprising weight (kg), length (cm), and head circumference (cm) as well as BMI were collected at the same time as the breast milk sample.

### 2.2. Leptin Measurement

Samples were thawed on ice for 2 h and continuously vortexed during pipetting to maintain homogeneity. Leptin concentrations (ng/mL) were quantified in all samples. Skim milk was obtained by centrifugation of 200 µL of whole milk at 12,000× *g* for 20 min at 4 °C, followed by removal of the fat layer. The resulting fat-free (skim) fraction was analysed using a commercial ELISA kit (R&D Systems™ Human Leptin Quantikine ELISA Kit, Minneapolis, MN, USA). All assays were performed in a single laboratory by the same technician to ensure consistency.

### 2.3. Statistical Analysis

Statistical analyses were performed using R version 4.3.1 (R Foundation for Statistical Computing, Vienna, Austria; https://www.R-project.org) and Matlab R2023a. Differences between delivery types were assessed using Wilcoxon post hoc analysis. Comparisons across BMI categories, physical activity levels, parity, lactation time and maternal age were evaluated using Bonferroni-adjusted analyses. Associations between leptin concentrations and clinical variables were examined using Spearman’s rank correlation coefficient. Statistical significance was set at *p* < 0.05, except for multiple group comparisons, where significance thresholds were adjusted according to the Bonferroni correction (*p* < 0.05/number of comparisons).

### 2.4. Linear Regression Analysis

All available maternal variables were initially considered in an exploratory model to evaluate their association with leptin levels. Variables that did not show statistical significance were removed stepwise to construct the final model, which included maternal BMI, physical activity, and delivery type (term vs. preterm) as predictors. Leptin levels were expressed in ng/mL. Model assumptions of linearity, normality, and homoscedasticity of residuals were verified and met. Of the initial 262 participants, only 91 were included in the final linear regression analysis due to incomplete data for one or more key maternal or neonatal variables. Missingness primarily affected maternal BMI, parity, physical activity, and neonatal anthropometric measures. Participants included in the regression analysis did not differ significantly from those excluded in terms of maternal age, delivery type, parity distribution, or neonatal characteristics, suggesting that the final analytical sample is largely representative of the full cohort. Although the exclusion of participants with missing values may reduce statistical power, it is unlikely to substantially bias the observed associations.

To assess potential multicollinearity, variance inflation factors (VIF) and correlation matrices were calculated for all predictors.

## 3. Results

### 3.1. Study Population

Table 1 summarizes the demographic and clinical characteristics of the participating mothers, including the distribution of samples across lactation stages. The mean maternal age was 33.8 years (SD: 4.4), ranging from 16 to 44 years. The majority of mothers (63.4%) were between 25 and 35 years old, 32.3% were older than 36, and only 4.3% were younger than 25.

Regarding nutritional status, the mean BMI at the time of sample collection was 24.7 kg/m^2^ (SD: 4.0). Half of the mothers (50.5%) had a BMI within the normal range (18.5–24.9 kg/m^2^), while 18.3% and 19.3% were classified as overweight, with BMI values of 25–26.9 and 27–29.9, respectively. Obesity (BMI ≥ 30) was observed in 11.9% of the participants.

In terms of lifestyle, 44.9% of the mothers reported low levels of physical activity, 37.4% engaged in moderate activity, and 10.2% were considered highly active. A small proportion (7.5%) reported a sedentary lifestyle.

With respect to obstetric history, 58.6% of the mothers had full-term deliveries, while 41.4% delivered preterm. More than half (54.6%) were primiparous, 28.1% had two deliveries, and 17.3% reported more than three deliveries.

### 3.2. Variations in Leptin Concentrations

**Figure 1:**
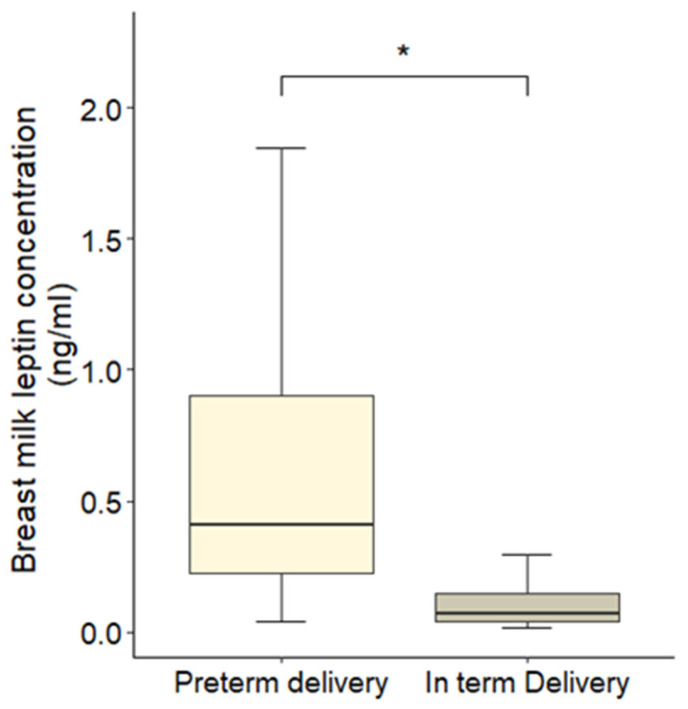
Differences in HBM leptin concentrations between preterm and term deliveries. Data are presented as mean ± SD, with error bars representing SD. * indicates a statistically significant difference between groups (*p* < 0.001, Wilcoxon post hoc analysis).

Leptin concentrations in HBM varied significantly according to delivery type. Mothers who delivered preterm exhibited markedly higher leptin levels compared to those with term deliveries (mean ± SD: 0.42 ± 0.15 vs. 0.07 ± 0.03 ng/mL; *p =* <2.2 × 10^−16^) (Figure 1).

**Figure 2:**
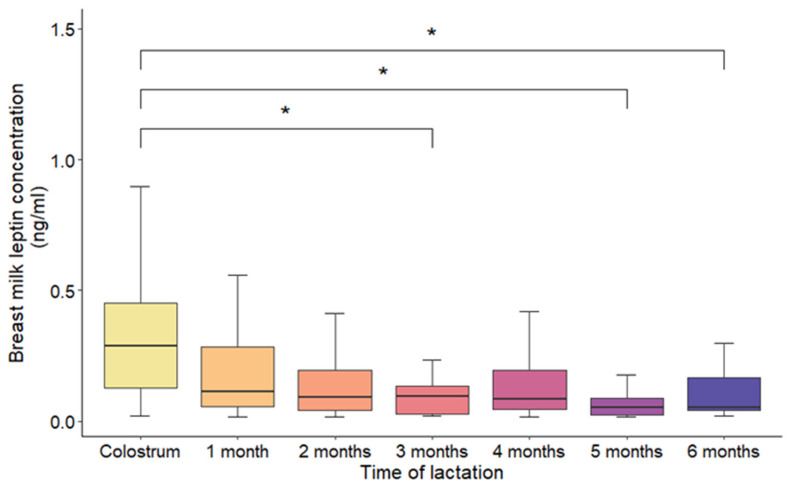
Leptin concentration (ng/mL) differences during the time of lactation. Data are presented as mean ± SD, with error bars representing SD. * indicates a statistically significant difference between groups (*p* < 0.01; Bonferroni adjusted test).

Analysis of leptin concentrations across lactation stages revealed the highest levels in colostrum (0.32 ± 0.12 ng/mL), followed by a significant decline by one month postpartum (0.11 ± 0.05 ng/mL; *p* < 0.01). From the second month onward, leptin concentrations remained low and stable, with no significant differences observed between months 2 and 6 (range: 0.05–0.07 ng/mL) (Figure 2).

**Figure 3:**
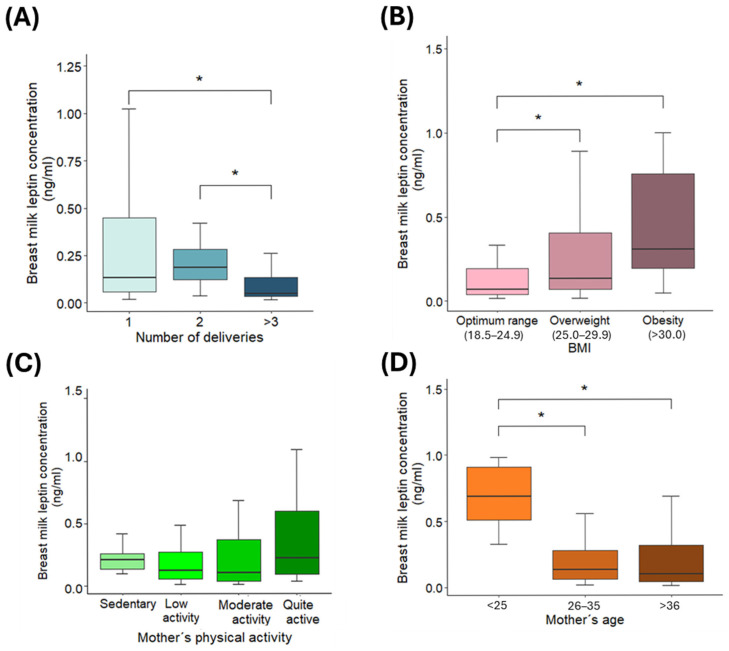
Leptin concentrations (ng/mL) in human breast milk according to: (**A**) number of deliveries, (**B**) lactation stage up to 6 months, (**C**) maternal physical activity level, and (**D**) maternal age. Data are presented as mean ± SD, with error bars representing SD. * indicates a statistically significant difference between groups (*p* < 0.01; Bonferroni adjusted test).

Parity was also associated with leptin levels in HBM. Mothers with a single delivery or 2 deliveries had significantly higher concentrations than those with three or more deliveries (0.17 and 0.19 ng/mL vs. 0.05 ng/mL; *p* < 0.01) (Figure 3A). Moreover, maternal BMI showed a positive correlation with leptin concentrations: obese mothers (BMI ≥ 30 kg/m^2^) had the highest levels (0.36 ± 0.10 ng/mL), followed by overweight mothers (BMI 25.0–29.9 kg/m^2^) (0.13 ± 0.06 ng/mL), while mothers with normal BMI (18.5–24.9 kg/m^2^) exhibited the lowest concentrations (0.05 ± 0.02 ng/mL) (Optimum range vs. overweight *p* = 0.0178; Optimum range vs. obesity *p* = 0.0017) (Figure 3B).

On the other hand, no significant differences were found in leptin concentrations based on maternal physical activity levels, indicating that this variable does not appear to influence HBM leptin content (0.23 ng/mL in sedentary mothers versus 0.13 ng/mL in mothers with low and moderate activity versus 0.29 ng/mL in fairly active mothers) (Figure 3C). In contrast, maternal age was significantly associated with leptin levels: mothers younger than 25 years showed substantially higher concentrations compared to older age groups (0.80 ± 0.20 vs. 0.10 ± 0.04 ng/mL; *p* = 0.0007 in both comparisons) (Figure 3D).

Finally, a strong inverse correlation was observed between leptin concentrations in HBM and newborn anthropometric parameters. Higher newborn weight, length, and head circumference were consistently associated with lower leptin levels in breast milk. All correlations were statistically significant (*p* < 0.0001) (Table 2).

### 3.3. Linear Regression Model

Linear regression analysis was conducted to examine the associations between leptin levels and mother characteristics. The model included 91 participants after excluding missing data. An initial model was conducted which included all available maternal variables. Non-significant predictors were subsequently removed through a stepwise procedure, yielding the final model comprising maternal body mass index (BMI), physical activity and birth term group.

The results are summarized in Table 3. BMI was positively associated with leptin levels (β = 0.031, *p* = 0.001), indicating that higher BMI corresponds to higher leptin concentrations. Additionally, participants in preterm group showed higher leptin levels compared with in term group (β = 0.524, *p* < 0.001). Although physical activity was not significantly associated with human breast milk leptin levels in the univariate analyses, it reached statistical significance in the multivariate regression model. This likely reflects adjustment for confounding by maternal BMI and birth term, which allowed the independent contribution of physical activity to emerge. To assess potential multicollinearity, variance inflation factors (VIF) were calculated for all predictors included in the final model (Appendix A. Table A1). All VIF values were below 2 (BMI: 1.02; physical activity: 1.03; birth term: 1.07), indicating minimal collinearity. Therefore, the observed effect of physical activity is unlikely to result from correlated predictors or modelling artifacts, supporting its interpretation as an independent factor associated with leptin concentrations in breast milk.

Overall, the model explained 57.9% of the variance in leptin levels (R^2^ = 0.579, Adjusted R^2^ = 0.554; F-statistic: 23.36 on 5 and 85 DF, *p*-value: 1.075 × 10^−14^), with a residual standard error of 0.338 ng/mL. Increasing the sample size or including additional complete observations could further enhance the robustness and precision of the model estimates.

## 4. Discussion

This study examined the relationships between maternal characteristics, neonatal and leptin concentrations in human breast milk (HBM). Leptin, primarily produced by adipose tissue, is also synthesized in the placenta [23] and mammary gland, making it an intrinsic component of breast milk [16,24]. Its presence in HBM has prompted growing interest due to its potential role in infant growth regulation, metabolic programming, and long-term obesity risk [16,24].

Our findings reinforce the notion that maternal BMI is a major determinant of HBM leptin concentrations. Higher BMI was associated with elevated leptin levels, consistent with previous evidence linking maternal adiposity to breast milk composition and its impact on the infant’s metabolic outcomes [15,16,17,18,25,26,27,28]. This observation is further supported by a recent systematic review by Qureshi et al. [14], which analyzed 33 studies and reported a consistent positive association between maternal adiposity—assessed as BMI or body weight either pre-pregnancy or during lactation—and breast milk leptin concentrations. Importantly, this review also highlighted substantial heterogeneity across studies and emphasized that BMI should be interpreted with caution, as it is an indirect and imperfect proxy of adiposity and metabolic status rather than a direct measure of maternal fat mass or endocrine function. It is important to note that BMI was assessed during lactation rather than pre-pregnancy or at delivery, which constrains its validity as a surrogate marker of gestational adiposity.

However, the biological implications remain unclear. While leptin may contribute to appetite regulation and energy balance [29,30], other study suggests possible adverse metabolic effects [31], underscoring the need for cautious interpretation and consideration of additional hormonal and nutritional factors beyond BMI. In this context, emerging evidence suggests that leptin may exert some of its effects through epigenetic mechanisms, rather than acting solely as an acute regulator of appetite or energy balance [32].

Delivery type emerged as a significant factor influencing leptin concentrations in HBM. Preterm milk contained markedly higher leptin levels than term milk. While this observation could reflect a potential compensatory or adaptive mechanism to support the metabolic needs and developmental requirements of premature infants, this hypothesis remains speculative and should be interpreted with caution. Although the literature presents mixed findings—with some studies reporting lower leptin concentrations in preterm milk [33], while others observe no notable differences [34,35]—our results are consistent with evidence suggesting that higher leptin in preterm milk may play a role in neonatal development, but the precise biological function and causality remain uncertain. This interpretation is in line with the study of Chatmethakul et al., who proposed that increased leptin in preterm milk reflects an adaptative response to neonatal metabolic immaturity [36]. Furthermore, the hormonal shifts associated with preterm birth, which influence both maternal and infant endocrine profiles, may contribute to the variability in leptin concentrations reported across studies [24,37,38,39,40]. Together, these endocrine dynamics highlight the complexity of lactational biology and its capacity to respond to the specific physiological challenges imposed by preterm delivery [34,35]. In this context, our multivariate analysis underscores that preterm birth itself emerges as an independent determinant of leptin levels in human milk, reinforcing the notion that lactation adapts hormonally to the demands of premature neonates.

Parity and maternal age were additional determinants. Primiparous mothers exhibited higher leptin levels than multiparous mothers, suggesting that hormonal or glandular changes across successive pregnancies may modulate leptin secretion [19,41,42]. Additionally, younger maternal age was associated with elevated leptin concentrations, consistent with previous reports [19,41]. However, the number of participants in the <25 years age group was small (n = 8) represents a significant limitation for the interpretation of these findings. The higher leptin levels observed in this subgroup may have been influenced by a limited number of extreme values and may not be representative of the broader population of younger mothers. In addition, the biological mechanisms underlying age-related differences in HBM leptin concentrations remain poorly understood, and current evidence is insufficient to explain why younger mothers might exhibit higher leptin levels. These findings indicate that reproductive history and maternal age are not merely demographic descriptors but may actively shape the hormonal profile of breast milk, with potential implications for infant development.

In contrast, maternal physical activity did not show a significant association with HBM leptin concentrations. Although a non-significant trend toward higher leptin levels with increased activity was observed, our results support the hypothesis that mammary leptin synthesis may be relatively independent of maternal activity levels [17,42]. It should be noted that maternal physical activity was evaluated using categorical self-report rather than a validated instrument, which may limit measurement accuracy and the interpretability of its association with human breast milk leptin concentrations.

Maternal physical activity did not show a significant association with HBM leptin concentrations, contrary to what the multivariate model might initially suggest. Although a non-significant trend toward higher leptin levels with increasing activity was observed and some model coefficients reached statistical significance, the lack of a consistent dose–response pattern suggests that the associations observed in the model may reflect residual confounding or the influence of correlated maternal characteristics (e.g., maternal age, BMI) rather than a causal effect of physical activity itself. Overall, the apparent discrepancy between univariate and multivariate results indicates that maternal physical activity does not exert a robust or biologically consistent influence on HBM leptin concentrations within the present dataset, supporting the hypothesis that mammary leptin synthesis could be independent of maternal activity levels [17,42]. Importantly, this discrepancy cannot be fully resolved within the current dataset and is therefore acknowledged as a methodological limitation of the study.

Significantly, we identified a strong inverse correlation between leptin concentrations in HBM and neonatal anthropometric measures, including weight, length, and head circumference. This pattern suggest that elevated leptin levels may act as a signal of energy sufficiency, potentially influencing infant appetite regulation and growth dynamics [18,43,44,45,46,47], although the directionality and underlying mechanisms remain unclear [48]. From a mechanistic perspective, BM leptin could influence infant outcomes through epigenetic pathways, including alterations in DNA methylation, histone modifications, and non-coding RNA expression, particularly during early postnatal life when epigenetic plasticity is high. As reviewed by Wróblewski et al. [32], leptin-related epigenetic regulation has been implicated in metabolic disorders and may represent a plausible biological link between early hormonal exposure and long-term metabolic programming.

While some studies support leptin’s role as a satiety hormone or regulator of energy expenditure [49,50], others report inconsistent or null associations with infant growth metrics [51]. Importantly, the possibility of reverse causation should be considered. Smaller or less mature infants may influence breast milk composition through altered suckling patterns, feeding frequency, or maternal physiological responses, potentially contributing to higher leptin concentrations in milk. Therefore, the observed associations cannot be interpreted as evidence of a unidirectional effect of HBM leptin on neonatal growth. This heterogeneity underscores the complexity of leptin’s function in early development and highlights the need for further investigation.

It should be noted that these correlations are unadjusted and do not account for potential confounding maternal or neonatal factors. Multivariate neonatal models would be required to determine whether these associations are independent. Future studies with larger sample sizes and comprehensive neonatal modeling are warranted to clarify the potential causal role of HBM leptin in shaping neonatal anthropometric outcomes.

Supporting this view, several studies have reported inverse relationships between HBM leptin and infant anthropometric parameters, including weight [25,41,52], BMI [19,26,53] and early postnatal weight gain [50]. These associations may persist beyond the neonatal period, with some evidence suggesting sustained effects up to 6 months [25], 1 year [41], or even 2 years of age [54]. However, other investigations have found no association with infant BMI [17], underscoring the heterogeneity of results across populations and study designs.

Taken together, these observations support the hypothesis that leptin may act as a regulatory signal in infant growth, potentially influencing energy allocation and fat deposition during critical developmental windows. Nonetheless, the biological mechanisms underlying these associations remain poorly understood. Longitudinal studies with standardized methodologies and comprehensive maternal-infant profiling are essential to clarify leptin’s role in early metabolic programming.

Our findings reinforce the intricate relationship between maternal characteristics and leptin concentrations in HBM. Elevated leptin levels observed in colostrum and preterm milk may represent adaptive physiological mechanisms designed to support neonatal development during critical early stages. These adaptations appear particularly relevant for preterm infants, whose metabolic systems are still maturing.

Key maternal factors—BMI, parity, and age—were significantly associated with variations in HBM leptin levels, underscoring the pivotal role of maternal health and physiology in shaping HBM composition. These associations suggest that maternal nutritional status and metabolic health directly influence the bioactive profile of breast milk, thereby affecting infant nutritional exposures and developmental trajectories.

The linear regression analysis showed that BMI and preterm delivery were positively associated with leptin levels, whereas higher physical activity was associated with lower leptin concentrations. The positive relation between adiposity (as reflected by BMI) and leptin is well established; during pregnancy, higher maternal BMI has been consistently linked to elevated circulating leptin levels [55,56]. Likewise, our finding that term delivery influences leptin levels aligns with evidence that maternal metabolic and gestational context—including gestational age or pregnancy related physiological changes—can modulate leptin synthesis [55]. In contrast, the inverse association with physical activity is consistent with studies showing that exercise or increased energy expenditure may reduce leptin concentrations, likely through effects on adipose tissue metabolism and overall energy balance [56,57]. Although lactation stage was described in the results, it was not included in the final multivariable model because it showed no significant independent association with leptin levels. However, residual confounding cannot be ruled out, particularly since some mothers provided samples at different lactation times. This should be considered a study limitation, and future longitudinal research should examine the role of lactation stage more thoroughly.

Our model explained 57.9% of the variance in leptin, indicating a solid fit for biological data. Nevertheless, a larger sample size or more complete observations would likely improve the precision and robustness of these estimates. Overall, these results highlight the relevance of maternal anthropometry, gestational context, and lifestyle behaviors in shaping leptin regulation during pregnancy.

This study contributes to a growing body of literature recognizing leptin as a vital bioactive component in early-life nutrition. Our results support the hypothesis that optimizing maternal health through targeted nutritional, lifestyle, and clinical interventions may enhance HBM quality and promote favorable outcomes in infant growth and metabolic programming. Future research should aim to integrate maternal metabolic profiles, infant feeding behaviors, and long-term developmental metrics to elucidate the broader implications of leptin signaling during infancy.

Compared to previous investigations, our study benefits in general from a larger sample size and the inclusion of diverse maternal and neonatal variables, allowing for a more comprehensive analysis of leptin dynamics in HBM. These findings corroborate and extend existing evidence on leptin’s role in early metabolic development, offering new insights into the maternal and environmental determinants of breast milk composition.

Nonetheless, certain limitations must be acknowledged. A limitation is the absence of detailed data on breast milk expression modalities—such as total volume expressed, or the time of day of expression—since the samples were obtained from human milk biobanks rather than standardized expression sessions. Although sample sizes at each lactation stage were sufficient for robust statistical analysis, longitudinal data were not available for all participants across every time point. This partial sampling substantially limits the interpretation of the observed decline in leptin over time, as within-subject changes cannot be robustly evaluated. In addition, BMI was measured during lactation rather than pre-pregnancy or at delivery, limiting its usefulness as a proxy for gestational adiposity. Other limitations include the reduced sample size for regression analyses (n = 91) and the use of a non-validated assessment of maternal physical activity. Future studies with complete longitudinal follow-up and standardized methodologies are essential to validate and expand upon these findings.

## 5. Conclusions

In summary, our results highlight the multifactorial nature of leptin regulation in HBM as well as its associations with infant growth and long-term metabolic outcomes. Increased leptin concentrations in colostrum and preterm milk may reflect adaptive responses to developmental demands and highlight the adaptive hormonal adjustments that support the metabolic vulnerability of premature infants. Maternal BMI, parity, and age emerge as maternal characteristics associated with variations in breast milk composition. Continued investigation into leptin’s role in early-life nutrition, using longitudinal and mechanistic approaches, will be critical for informing maternal health strategies and optimizing infant development.

## Figures and Tables

**Table 1:** Characteristics of the mothers and breast milk samples included in the study. Data are presented as n (%).

Variable	Category	n (%)
Age (years)	<25	8 (4.3)
	25–35	118 (63.4)
	>36	60 (32.3)
BMI (kg/m^2^)	18.5–24.9	55 (50.5)
	25–29.9	41 (37.6)
	>30	13 (11.9)
Physical activity	Sedentary	11 (7.5)
	Low activity	66 (44.9)
	Moderate activity	55 (37.4)
	Quite active	15 (10.2)
Delivery type	Term	109 (58.6)
	Preterm	77 (41.4)
Number of deliveries	1	101 (54.6)
	2	52 (28.1)
	≥3	32 (17.3)
Lactation stage	Colostrum	36 (13.7)
	1 month	107 (40.9)
	2 months	23 (8.8)
	3 months	33 (12.6)
	4 months	21 (8.0)
	5 months	10 (3.8)
	6 months	32 (12.2)

**Table 2:** Spearman’s correlation coefficients between leptin concentration and newborn anthropometric measures.

	Spearman’s ρ	*p* Value
Newborn weight	−0.582	8.05 × 10^−17^
Newborn length	−0.576	1.59 × 10^−15^
Head circumference	−0.562	3.51 × 10^−14^

Spearman’s ρ = Spearman correlation coefficient. All correlations were significant at *p* < 0.0001.

**Table 3:** Multiple linear regression model predicting leptin levels Model fit: R^2^ = 0.579; adjusted R^2^ = 0.554; F(5, 85) = 23.36; *p* < 0.001.

Predictor	β	SE	95% CI	t	*p* Value
Interceptor	1.291	0.360	0.585 to 1.997	3.59	<0.001
BMI (kg/m^2^)	0.031	0.009	0.013 to 0.049	3.40	0.001
Physical activity					
Low activity	−1.862	0.247	−2.346 to −1.378	−7.53	<0.001
Moderate activity	−1.952	0.250	−2.442 to −1.462	−7.80	<0.001
Quite active	−1.820	0.267	−2.343 to −1.297	−6.83	<0.001
Gestational age (ref: Term group)					
Preterm group	0.524	0.100	0.328 to 0.720	5.23	<0.001

Abbreviations: β, regression coefficient; SE, standard error; CI, confidence interval. Reference categories are indicated in parentheses.

## Data Availability

Data are not publicly available due to privacy and ethical restrictions approved by the Ethics Committee (57/723952.9/22) and Scientific Committee (PI22/332).

## Abbreviations

The following abbreviations are used in this manuscript:

HBM	Human Breast Milk
BMI	Body Mass Index

## Appendix A

**Table A1:** Assessment of multicollinearity among predictors included in the linear regression model.

Predictor Variable	Generalized Variance Inflation Factor (GVIF)	Degrees of Freedom (df)	GVIF1/(2·df)
BMI (kg/m^2^)	1.05	1	1.02
Physical activity	1.20	3	1.03
Delivery type (term/preterm)	1.15	1	1.07
